# Efficacy of Corticosteroid Therapy on Neurological Outcomes in Near-Hanging Patients With Altered Sensorium: Protocol for a Multicenter, Double-Blinded, Placebo-Controlled, Randomized Trial (CHANGING Trial)

**DOI:** 10.2196/100623

**Published:** 2026-08-25

**Authors:** Ramadoss Ramu, Dineshbabu Sekar, Aswin K, Ashish Behera, Manu Ayyan, Harichandra Kumar KT, Kavitha B, Mohan Kumar Hanumanthappa, Sree Naga Sowndary Rajendran

**Affiliations:** 1 Medicine, Associate Professor, Jawaharlal Institute of Post Graduate Medical Education and Research, Department of Medicine, Pondicherry, Puducherry, 605006, India, 91 9582318223; 2 Emergency Medicine and trauma, Assistant Professor, Indira Gandhi Medical College and Research Institute, Pondicherry, Puducherry, India; 3 Medicine, Associate Professor, Post Graduate Institute of Medical Education and Research, Chandigarh, Chandigarh, India; 4 Emergency Medicine and trauma, Associate Professor, Jawaharlal Institute of Post Graduate Medical Education and Research, Pondicherry, Puducherry, India; 5 Biostatistics, Assistant Professor, Jawaharlal Institute of Post Graduate Medical Education and Research, Pondicherry, Puducherry, India; 6 Medicine, Professor, Indira Gandhi Medical College and Research Institute, Pondicherry, Puducherry, India; 7 Medicine, Project Research Scientist, Jawaharlal Institute of Post Graduate Medical Education and Research, Pondicherry, Puducherry, India

**Keywords:** near hanging, dexamethasone, corticosteroid, cerebral edema, Glasgow Outcome Scale

## Abstract

**Background:**

In India, hanging is the leading method of suicide, and survivors of near-hanging often present with altered sensorium due to cerebral edema from cervical vascular compression and hypoxia. Management typically involves supportive care and antiedema measures, such as mannitol, hypertonic saline, and dexamethasone. Dexamethasone, a corticosteroid that crosses the blood-brain barrier, is commonly used for presumed cerebral edema. However, evidence supporting its efficacy is limited, and there are no clinical guidelines that recommend its routine use. A randomized controlled trial is therefore essential to establish the real-world efficacy of dexamethasone in this setting.

**Objective:**

The primary objective of this study is to evaluate the efficacy of intravenous dexamethasone, compared with placebo, in achieving good neurological outcomes at day 28. Secondary objectives are to assess the efficacy of dexamethasone, compared with placebo, in improving neurological outcomes at hospital discharge. We will also compare the duration of hospital stay and the incidence of in-hospital complications between patients receiving dexamethasone and those receiving a placebo.

**Methods:**

The CHANGING trial (Corticosteroids in HANGING) is a multicenter, double-blind, randomized, placebo-controlled study at 3 Indian tertiary centers. It enrolls 192 near-hanging patients with a Glasgow Coma Scale score ≤13 over 3 years, randomizing them in a 1:1 ratio to IV dexamethasone or placebo, plus standard care for 3 to 5 days. The primary outcome is neurological recovery at day 28, assessed using the Glasgow Outcome Scale. Secondary outcomes include the Glasgow Outcome Scale at discharge, duration of hospital stay, and the incidence of in-hospital complications. This trial has been approved by Jawaharlal Institute of Postgraduate Medical Education and Research, Puducherry Institute Ethics Committee (January 22, 2025), with subsequent approvals from Indira Gandhi Medical College and Research Institute (April 8, 2025) and Postgraduate Institute of Medical Education and Research (August 26, 2025).

**Results:**

The study commenced in October 2025 and is planned to be completed by May 2028. It is currently ongoing, and patient recruitment is in progress. Results will be analyzed after completion of enrollment and follow-up and will be disseminated through peer-reviewed publications.

**Conclusions:**

Corticosteroids are widely used for cerebral edema after near-hanging, but their role is unclear. The CHANGING trial will test dexamethasone’s efficacy and safety in these patients to guide future practice and guidelines.

## Introduction

### Background

Suicide is a major global health issue, with over 700,000 deaths in 2019 [1]. In India, hanging accounts for 58.2% of suicides, making it the leading cause [2]. Fatal hanging causes immediate death, while nonfatal (or near-hanging) cases survive the attempt and manage to reach the hospital [3]. Patients surviving near-hanging often have varying degrees of injury to vital neck structures, often requiring intensive care. Key management focuses on airway protection, aspiration pneumonia, pulmonary complications, and cervical spine injury; preventing secondary brain injury caused by reperfusion after the release of the obstruction; and treating cerebral edema and hypoxic damage [4-8]. Neurological injury in the form of cerebral edema critically affects long-term outcomes [4,7,8].

Brain injury caused by near-hanging is due to cerebral hypoxia resulting from airway obstruction, compression of the carotid arteries, compression of the jugular veins, or a combination of these mechanisms. Secondary brain injury due to reperfusion further worsens brain damage [5,9]. The damage occurs through several processes, including inflammation, oxidative stress, damage to brain cells, and disruption of the blood-brain barrier, causing cerebral edema. Cerebral edema can further increase once compression is relieved and blood flow returns [10-12]. This cerebral edema may represent a therapeutic target, as it is a potentially reversible component of brain injury. This edema can be due to cytotoxic edema (cellular energy failure leading to intracellular water accumulation) or vasogenic edema (leakage of fluid into brain tissue after damage to the blood-brain barrier), and both types may occur together [10-13]. Although direct evidence in near-hanging is limited, these processes are well described in hypoxic brain injury, traumatic brain injury, ischemic stroke, high-altitude cerebral edema (HACE), and other neurocritical illnesses [14-17].

Corticosteroids, such as dexamethasone, are favored for their blood-brain barrier penetration and reduce vasogenic cerebral edema by stabilizing the blood-brain barrier, reducing endothelial permeability, and suppressing inflammation [14,18]. They are effective in conditions such as brain tumors and bacterial meningitis, but their role in acute brain injury remains doubtful. The Medical Research Council CRASH (Corticosteroid Randomization after Significant Head Injury) trial demonstrated increased mortality in traumatic brain injury with the use of corticosteroids [19].

Corticosteroids are used to treat cerebral edema in near-hanging patients with altered sensorium, despite limited evidence [20]. Their proven role in HACE, involving hypoxia- and inflammation-driven edema, prompts extrapolation to hanging cases [15-17]. As the pathophysiology of hanging-induced cerebral edema is poorly understood, this analogy is theoretical.

A retrospective study from a South Indian tertiary care center admitted 323 near-hanging patients over 5 years, most of whom were young-or middle-aged men. Steroids were given to 68% (219/323) of patients, mainly dexamethasone (200/219) [17]. Among patients with Glasgow Coma Scale (GCS) ≤13 cases (n=201), steroid recipients (n=160, 80%) had 77% good outcomes vs 90% in nonrecipients (n=41, 20%), but selection bias favored administering steroids to sicker patients, and steroid dosing and duration varied. No established guidelines exist for managing near-hanging, including steroid use, which contributes to wide practice variations [21].

### Study Rationale

At present, the use of corticosteroids in near-hanging patients with impaired consciousness is inconsistent and guided largely by individual clinicians’ preferences and assumptions about shared pathogenesis with other traumatic brain injuries and similar conditions, rather than by evidence-based recommendations. There are no established guidelines or randomized controlled trials addressing this therapeutic dilemma. This lack of clarity creates genuine clinical equipoise regarding the role of steroids in managing cerebral edema following near-hanging, underscoring the urgent need for high-quality prospective evidence.

### Expected Outcome

This study aims to systematically evaluate the efficacy of steroid therapy in near-hanging patients with altered sensorium. The findings are expected to generate evidence that may guide clinical decision-making and contribute to the development of standardized treatment recommendations.

### Objectives

The primary objective of this study is to evaluate the efficacy of intravenous dexamethasone, compared with placebo, in achieving a good neurological outcome (Glasgow Outcome Scale [GOS] 4‐5) at day 28 in patients presenting with near-hanging with altered sensorium. Secondary objectives are to assess neurological outcomes at hospital discharge, compare the length of hospital stay, and compare the incidence of in-hospital complications between the dexamethasone and placebo groups.

## Methods

### Overview

**Figure 1.**
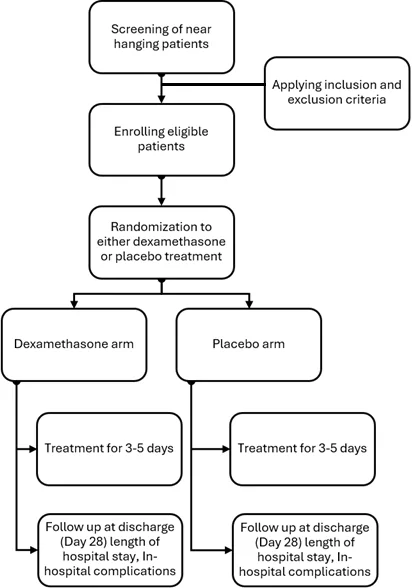
Flow chart depicting the various stages of the study.

The CHANGING trial (Corticosteroids in HANGING) is a multicenter, double-blind, randomized, placebo-controlled clinical trial designed to evaluate whether intravenous dexamethasone improves neurological outcomes in patients presenting with near-hanging. This protocol has been designed in accordance with the SPIRIT (Standard Protocol Items: Recommendations for Interventional Trials) 2013 statement (Checklist 1) to ensure robust and transparent reporting of the study methodology [22,23]. The flow chart depicting the various stages of the study is shown in Figure 1.

### Study Organization

The primary site, Jawaharlal Institute of Postgraduate Medical Education and Research (JIPMER), Puducherry, is the coordinating site. The principal investigator and coinvestigators at the primary site were involved in protocol development and securing funding for this trial from the Indian Council of Medical Research (ICMR), New Delhi. The primary site will conduct training, data monitoring, randomization, and quality control for the other 2 sites, Indira Gandhi Medical College and Research Institute (IGMCRI), Puducherry, and Postgraduate Institute of Medical Education and Research (PGIMER), Chandigarh. All 3 sites will competitively recruit consecutive patients admitted with near-hanging. All sites are responsible for following the protocol and ensuring the quality of the data collected.

### Study Setting

This trial will be conducted in the Departments of Medicine and Emergency Medicine at 3 tertiary care centers in India: JIPMER, Puducherry; IGMCRI, Puducherry; and PGIMER, Chandigarh.

### Study Participants

Patients with near-hanging presenting to the emergency medicine departments of 3 study centers—JIPMER, Puducherry (JI); IGMCRI, Puducherry (IG); and PGIMER, Chandigarh (PG)—will be the study participants.

### Eligibility Criteria

The inclusion and exclusion criteria for the study participants are shown in Textbox 1.

Textbox 1. **Inclusion and exclusion criteria for study participants.**

**Inclusion criteria**

- Age ≥18 years.
- History of near-hanging.
- Altered sensorium with Glasgow Coma Scale (GCS) ≤13 at presentation.
- Presentation to the hospital within 48 hours of the hanging event.

**Exclusion criteria**

- Concurrent use of other methods of suicide.
- Active tuberculosis, HIV infection, recent systemic steroid use, other immunosuppressive therapy, or any definite indication or contraindication to corticosteroid therapy.
- Known hypersensitivity to dexamethasone or study-related drugs.
- Pregnancy or breastfeeding.
- Pre-existing neurological disorders.
- Hanging-related cervical spine injury.
- Moribund state where survival is deemed unlikely irrespective of intervention.

### Randomization and Allocation Concealment

Central randomization will be conducted at the coordinating center. Participants will be randomly allocated in a 1:1 ratio to receive either intravenous dexamethasone or placebo using a computer-generated randomization sequence. Variable block sizes of 4 and 6 will be used, with stratification based on baseline GCS score (≤8 and >8) to ensure balance between the treatment groups. Allocation concealment will be ensured using the Sequentially Numbered Opaque Sealed Envelopes method.

**Figure 2.**
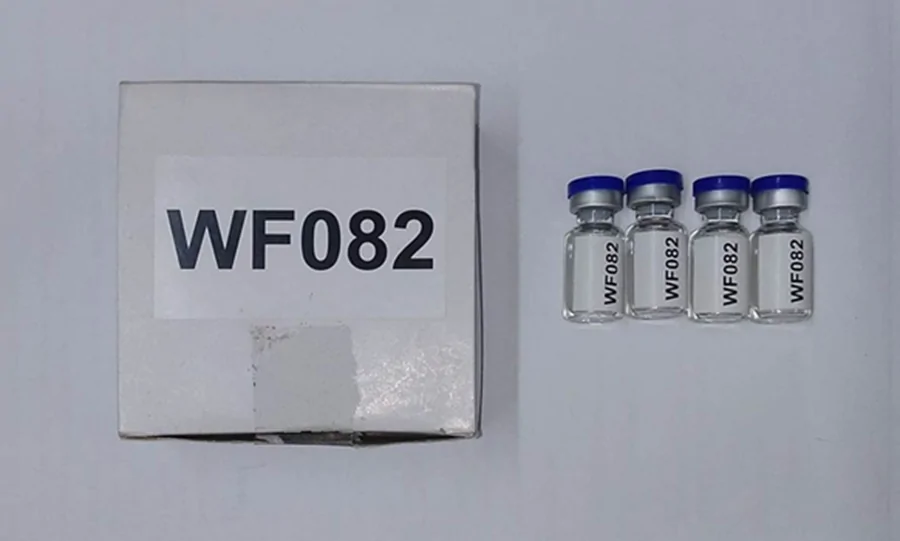
Medication kit. The left panel shows that a box is labeled with a randomly generated 5-character alphanumeric sequence. The right panel displays the sample medication kit with the same label.

The study medications (dexamethasone or placebo) are prepared in identical-looking vials and labeled with a 5-character alphanumeric code using a randomly generated sequence, as shown in Figure 2. They are securely packed in designated boxes for each site. The random sequence used for labeling the study medications is stored in a password-protected Microsoft Excel sheet, accessible only to an unblinded investigator at the primary site to ensure confidentiality and maintain blinding throughout the study.

A designated coinvestigator who is the only unblinded person other than the study statistician at the primary site will assume responsibility for crucial aspects of randomization, allocation concealment, and maintenance of the blinding process. All other staff (investigators, nurses, outcome assessors, and research team) and participants remain blinded to treatment assignments throughout the trial. After eligibility confirmation and written consent, the site team contacts the unblinded coinvestigator at the coordinating center. This investigator reconfirms eligibility and consent, performs randomization, and shares only the study drug label number supplied to each site, never revealing the actual treatment allocation.

### Trial Treatment

#### Study Interventions

All patients will receive standard medical care for near-hanging. Standard medical care includes head-end elevation, anticerebral edema measures using either mannitol or hypertonic saline, mechanical ventilator support (if required), and vasopressor support (if required)

Participants will be treated with the IV study drug for a minimum period of 3 days and a maximum of 5 days. The study drug can be stopped after a minimum of 3 days of therapy if the patient’s sensorium has improved to a GCS score of 15. Tapering of steroids (or placebo) is not required, as the duration of therapy is short. If steroids are stopped but cerebral edema causes a worsening of sensorium, steroids can be restarted, and this will be recorded.

#### Trial Treatment Schedule

This clinical trial compares 3 to 5 days of treatment with IV dexamethasone 8 mg stat, followed by 4 mg every 6 hours daily vs placebo, as shown in Textbox 2. This dose is adapted from the HACE treatment protocol, in which hypoxia similarly leads to cerebral edema [15].

Textbox 2. **Trial treatment schedule**

**Day 1**

- Dexamethasone arm: dexamethasone 8 mg IV administered as a slow IV push stat followed by 4 mg every 6 hours.
- Placebo arm: identically looking placebo of similar volume administered slow IV push similar to the dose of dexamethasone.

**Day 2 and 3**

- Dexamethasone arm: dexamethasone 4 mg every 6 hours.
- Placebo arm: placebo IV every 6 hours.

**Day 4**

- Assessment on day 4 to determine whether dexamethasone or placebo should be continued (based on GCS).

**Day 4-5 assessment**

- Dexamethasone arm: dexamethasone 4 mg IV every 6 hours.
- Placebo arm: placebo IV every 6 hours.

#### Premature Discontinuation of Randomized Treatment

The planned duration of study drug administration is 3 to 5 days. Earlier neurological recovery (GCS 15/15) is expected in some participants; a predefined, protocol-specified tapering or discontinuation strategy will be followed to minimize unnecessary drug exposure, as shown in Table 1.

**Table 1.** Premature discontinuation of randomized treatment.

Day when GCS^a^ 15/15 is first attained	Assessment time point	Study drug to be continued until
Day 1	6th, 12th, or 18th hour	Corresponding time points on day 2: 6th, 12th, or 18th hour (additional 24 hours)
Day 2	Any time point	Day 3
Day 3 or 4	Any time point	Based on clinical assessment by the study team
Day 5	–^b^	No further study drug will be administered

a GCS: Glasgow Coma Scale.

b Not applicable.

#### Mandatory Discontinuation

Study treatment will be permanently discontinued at any time if a participant develops a definite indication for or contraindication to dexamethasone or if discontinuation is clinically indicated, as determined by the treating investigator.

Premature discontinuation of study treatment will not affect protocol-mandated follow-up. All randomized participants will continue follow-up as per the approved study schedule.

### Investigational Product Management

#### Overview

The investigational products, dexamethasone and placebo, have been procured from a Good Manufacturing Practice (GMP)-certified pharmaceutical manufacturer, Jackson Laboratory Pharmaceutical Company, with dexamethasone supplied as a 4 mg/2 mL injectable solution in plain glass vials. The placebo consists of an equivalent volume (2 mL) of normal saline for injection, supplied in identical glass vials to ensure blinding.

A kit of study medication, comprising 22 vials for each patient, is assigned a unique 5-character alphanumeric identifier. The medication kit with the labeled vials is shown in Figure 2.

Manufacturing details, expiry dates, and recommended storage conditions have been communicated to investigators at all participating sites, and relevant documentation has been shared for regulatory and monitoring purposes.

Drug distribution is centrally coordinated by a designated coinvestigator at the coordinating center. The first batch of study medication has been supplied to all participating centers with sufficient buffer stock to support recruitment for the initial 2 years. Additional batches will be procured and distributed as needed, aligned with expiry timelines. Each site will maintain a drug accountability log documenting receipt, dispensing, and disposal. Unused or expired study medication will be discarded according to institutional biomedical waste management protocols.

#### Storage of Study Drugs

The investigational product and placebo will be stored at room temperature (20°C‐25 °C) in accordance with local institutional practices. Temperature monitoring will be performed when facilities are available; otherwise, the drugs will be stored at ambient room temperature under appropriate conditions to maintain stability.

### Data Collection and Management

Data will be collected by trained staff at each center using standardized case report form (CRF) booklets and entered into a password-protected REDCap (Vanderbilt University) electronic database. As each participant is given a unique enrollment ID, the data will be collected in a deidentified manner. Data will include demographics, medical history, comorbidities, details of the hanging event, and findings from a complete physical examination. Baseline investigations will comprise a complete hemogram, renal and liver function tests, serum electrolytes, random blood sugar, chest X-ray, ECG, and arterial blood gas analysis with PaO₂ and PaO_2_/FiO_2_ (PF) ratio. NCCT of the head and cervical spine will be performed when clinically indicated to evaluate for cerebral edema or cervical spine injury.

During the first 5 days of hospitalization, patients will undergo daily monitoring of physiological status, neurological function, organ dysfunction, and treatment variables. Neurological status will be assessed using the GCS at admission, every 6 hours on day 1, and every 24 hours thereafter, with adjustments for sedation. Hemodynamic parameters, including heart rate, mean arterial pressure, and the requirement for vasoactive agents, will be recorded, along with respiratory parameters such as respiratory rate, PF ratio, and ventilatory status. The need for organ support, including mechanical ventilation, inotropes, and sedatives, as well as details of antibiotic therapy, will be documented. Sequential Organ Failure Assessment (SOFA) scores will be calculated using platelet count, serum creatinine, bilirubin, mean arterial pressure or vasopressor requirement, PF ratio, and GCS at predefined time points.

Laboratory monitoring will be conducted to assess disease progression and potential steroid-related adverse effects. On days 2 and 3, a complete hemogram, blood glucose levels, and serum potassium will be measured to screen for complications such as infection, hyperglycemia, and hypokalemia. If steroid therapy is continued beyond day 3, similar laboratory monitoring will be repeated on days 4 and 5. SOFA scores will be recalculated on days 3 and 5. This monitoring schedule is shown in Table 2.

**Table 2.** Monitoring schedule (days 1‐5).

Domain	What is measured	Day 1	Day 2	Day 3	Day 4	Day 5
Neurological monitoring	GOS^f^ (adjusted for sedation)	At admission+every 6 hours	Every 24 hours	Every 24 hours	Every 24 hours	Every 24 hours
Hemodynamic parameters	Heart rate, MAP^b^, and vasopressor requirement	Daily	Daily	Daily	Daily	Daily
Respiratory parameters	Respiratory rate, PF^c^ ratio, and ventilatory status	Daily record	Daily	Daily	Daily	Daily
Organ support	Mechanical ventilation, inotropes, and sedatives	Daily	Daily	Daily	Daily	Daily
Antibiotic therapy	Type, dose, and duration	Baseline	—^e^	Record	—	Record
SOFA score components^d^	Platelets, creatinine, bilirubin, MAP or vasopressors, PF ratio, and GCS	Daily	Daily	Daily	Daily	Daily
Laboratory monitoring	Complete hemogram, blood glucose, and serum potassium	—	Check	Check	If on steroids → repeat	If on steroids → repeat
Purpose of laboratory monitoring	Detect infection, hyperglycemia, and hypokalemia	—	Check	Check	If on steroids → Check	If on steroids → Check

a GCS: Glasgow Coma Scale.

b MAP: mean arterial pressure.

c PF: PaO_2_/FiO_2_.

d SOFA (Sequential Organ Failure Assessment) scores will be calculated using platelet count, serum creatinine, bilirubin, MAP or vasopressor requirement, PF ratio, and GCS at predefined time points.

e Not applicable.

Patients will be closely observed for adverse events (AEs) throughout the study period, including nosocomial infections, significant gastrointestinal bleeding requiring transfusion, hyperglycemia (random blood sugar >180 mg/dL), electrolyte abnormalities, and cardiac or respiratory arrest requiring cardiopulmonary resuscitation. Protocol deviations, reasons for withdrawal, and final hospital outcomes will be documented. All participants will be followed up for 28 days, and for those discharged earlier, follow-up will be completed either through hospital visits or telephone contact if they are unable to visit the hospital. A screening log will be maintained at each participating center to document all patients admitted after a near-hanging.

AEs will continue to be monitored and documented. If participants continue to receive steroids for more than 5 days, this will be noted. All concomitant medications, including those given empirically to treat possible alternative causes of severe illness, will be recorded.

This study is prospective, so we do not expect any missing data. If we are unable to follow up and obtain outcome data, we will use imputation to fill in the missing data.

### Assessment at Discharge

The outcome assessment will include the GOS at discharge (shown in Table 3) [24]. We will also assess the duration of study medication, whether full sensorium is achieved during hospitalization, and the time taken to achieve full sensorium (defined as a GCS score of 15 maintained for more than 24 h). Additional outcomes will include the duration of hospital stay and intensive care unit stay, the need for ventilatory support, including its duration and type, and the requirement for vasoactive support and its duration, if applicable. All AEs, as defined in the study protocol, will be documented. For participants who do not complete the study, the reasons for noncompletion will be recorded in detail.

**Table 3.** Glasgow Outcome Scale (GOS) for neurological outcome [24].

GOS score	Rating	Description	Outcome category
1	Death	Death	Poor
2	Persistent vegetative state	Patient unresponsive and speechless for weeks or months	Poor
3	Severe disability	Dependent for daily support	Poor
4	Moderate disability	Disabled but independent	Good
5	Good recovery	Resumption of normal life with minor neurological and psychological deficits	Good

### Outcome Assessment on Day 28

The patient will be called for a follow-up on day 28 to assess GOS. The outcome will be assessed by the study staff who are blinded to the treatment allocation. If the patient cannot attend in person, GOS will be ascertained by telephone or video call. A standardized, structured interview process will be followed during the outcome assessment. Participants who die before day 28 will be assigned a GOS of 1 and will be included in the primary outcome analysis.

### Definition of the End of Study

The end of the study is defined as the time when the last participant has either completed the 28-day follow-up or discontinued the study early.

### Withdrawal Criteria

Each participant is free to discontinue participation or withdraw their consent at any time. If volunteered by the participant, the reason for discontinuation or withdrawal of consent will be recorded in the CRF. If a patient requests to stop participating, the investigator should ask if the patient would be willing to stop the treatment but continue follow-up. If they do not agree to this, the investigator should ask for their permission to contact them to determine their status on day 28.

### Rescue Criteria

Expected adverse effects of the intervention drug (dexamethasone) include hyperglycemia, hypokalemia, and infection. These adverse effects will be managed according to standard guidelines. If these adverse effects are not successfully treated, the participant will be withdrawn from the study, and the adverse effects will be managed.

### Safety Reporting

#### AE

An AE is any untoward medical occurrence in a participant to whom a medicinal product has been administered, including occurrences that are not necessarily caused by or related to that product. AEs are graded for severity as mild, moderate, severe, and life-threatening as shown in Table 4.

Expected adverse effects of the intervention drug (dexamethasone) are hyperglycemia, hypertension, gastritis, hypokalemia, and infection.

**Table 4.** Common Terminology Criteria for Adverse Events (CTCAE) [25].

Grade	Definition
Grade 1	Mild; asymptomatic or mild symptoms; clinical or diagnostic observations only; intervention not indicated.
Grade 2	Moderate; minimal, local or noninvasive intervention indicated; limiting age-appropriate instrumental ADL^a^^,^^b^.
Grade 3	Severe or medically significant but not immediately life-threatening; hospitalization or prolongation of hospitalization indicated; disabling; limiting self-care ADL^c^.
Grade 4	Life-threatening consequences, urgent intervention indicated
Grade 5	Death related to AE^d^

a ADL: activities of daily living.

b Instrumental ADL refer to preparing meals, shopping for groceries or clothes, using the telephone, managing money, and so on.

c Self-care ADL refer to bathing, dressing and undressing, feeding self, using the toilet, taking medications, and not bedridden.

d AE: adverse event.

#### Serious AE

A serious adverse event (SAE) is any untoward medical occurrence that (1) results in death, (2) is life-threatening, (3) requires inpatient hospitalization or prolongation of existing hospitalization (>21 d), (4) results in persistent or significant disability, or (5) involves any other important medical event that the investigator considers serious (eg, severe hyperglycemia causing diabetic ketoacidosis, gastrointestinal bleeding requiring blood transfusion, and infections causing septic shock requiring inotropic support).

#### Causality

The causality of each AE will be assessed by a medically qualified individual to determine its relationship to the trial medication. AEs will be categorized as definite when there is a clear-cut temporal association with the trial study drug. Events will be considered probable when there is a clear temporal association along with improvement after withdrawal of the drug and when the event cannot be reasonably explained by the patient’s known clinical condition or another etiology. Possible AEs will be those with a less clear temporal association, in which other etiologies could also account for the event; such alternative causes will be documented in the CRF. AEs will be classified as not related when there is no temporal association with the trial medication and the event is attributable to other causes, such as concomitant medications, underlying conditions, or the patient’s known clinical condition. The investigator will provide the assessment of causality as per the instructions for completion of the AE or SAE data collection tool.

#### Procedures for Recording AEs

Dexamethasone is an established and commonly used drug with a well-characterized toxicity profile. All AEs (grades 3‐4) will be recorded and reported. Mild and moderate AEs (grades 1‐2) will be recorded only if the investigator considers that the AE is definitely, probably, or possibly related to the study drug. All SAEs will be recorded and reported. If all the information required to report the event is not available initially, the SAE may first be recorded as an initial report, with follow-up and final reports recorded as needed. All AEs, serious and nonserious, will be recorded during the 28-day trial period.

The following information will be recorded: description, onset date and end date, severity, assessment of relatedness to the trial medication, other suspect drug or device, and action taken. Follow-up information should be provided as necessary. All AEs will be followed until resolution or until the event is considered stable.

The investigator will decide whether an AE is sufficiently severe to require the participant’s removal from treatment. A participant may also voluntarily withdraw from treatment because of what he or she perceives as an intolerable AE. If either of these occurs, the participant will undergo an end-of-trial assessment and receive appropriate care under medical supervision until symptoms cease, or the condition becomes stable.

All SAEs will be reported to the IEC within 24 hours of becoming aware of the SAE, and a detailed report will be submitted within 14 days of the event.

### Ethical Considerations

#### Screening, Informed Consent, and Enrollment

Study team members, trained at each site, will screen all near-hanging patients with altered sensorium presenting to participating emergency departments for eligibility. As these patients have impaired consciousness, informed consent will be obtained from their legally acceptable representative (LAR), who will receive a clear explanation of the study objectives, procedures, benefits, and risks and will have all questions answered before providing written consent, which will be countersigned by the investigator responsible for recruitment. Patients will be reconsented once they regain full consciousness (GCS 15/15). All screened patients will receive a unique screening ID, with exclusion reasons documented for noneligible cases; enrolled patients will receive a separate enrollment number for procedures and follow-up. To ensure standardized data tracking and patient confidentiality, a Unique Patient Identifier (UI) system will be used. Each center will use its 2-letter alpha code (JI, IG, or PG) followed by an “S“ for Screening or an “E“ for enrollment and then a 3-digit serial number (eg, 001, 002, and so on). For example, JIS006 identifies the sixth patient screened at the JIPMER site, while JIE003 identifies the third patient enrolled at the same site.

#### Ethical Approval

This clinical trial is conducted in accordance with the principles in the latest version of the Declaration of Helsinki. This trial was approved by the Institutional Ethics Committee of the lead site, JIPMER, Puducherry, India, dated January 22, 2025. The other sites subsequently obtained approval from their respective ethics committees (IGMCRI, Puducherry, India, on April 8, 2025, and PGIMER, Chandigarh, India, on August 26, 2025).

#### Ethical Issues

Patients or their attendants may be in a state of emotional distress after a suicide attempt. We will address this issue carefully. All participating sites submitted the study protocol, patient information sheet, and consent forms to their local ethics committee for approval. The patient information sheet and consent forms have been translated into the relevant regional languages and back translated into English to ensure the accuracy of the translation. Consent is initially obtained from the patient’s attendant, the LAR, as the patient will be unconscious. Later, once the patient regains consciousness, reconsent is obtained from the patient. The patient or the LAR is also given the opportunity to withdraw from the study at any time. The principal investigator has purchased clinical trial insurance using the allocated funds to ensure compensation for participants for any foreseeable and unforeseeable risks arising from the study.

### Trial Registration

This trial has been registered with the Clinical Trials Registry India (CTRI/2025/02/081343) on February 25, 2025. Patient recruitment commenced on October 3, 2025.

### Data Safety and Monitoring Board

Given the interventional nature of this trial, an independent Data and Safety Monitoring Board (DSMB) has been constituted in accordance with JIPMER’s DSMB guidelines to oversee participant safety and trial conduct. A DSMB charter was developed prior to trial initiation. The charter details the composition of the committee, minimum quorum requirements, frequency of meetings, the structure of meetings (open and closed sessions), and the procedures for documentation, communication, and reporting of recommendations.

The DSMB comprises 3 independent members: one expert from each of emergency medicine, internal medicine, and biostatistics. A quorum of 2 members is required for DSMB meetings. The study statistician will be responsible for presenting the interim efficacy analyses, and a consolidated summary of all AEs and SAEs will be submitted for DSMB review. The DSMB will periodically review accumulated safety and efficacy data, including a planned interim review after 50% enrollment in each treatment arm. Based on its evaluation, the DSMB may recommend continuation of the study without modification, protocol modification, or early termination of the trial. All DSMB recommendations will be formally communicated to the principal investigator, who will subsequently report them to the Institutional Ethics Committee.

An interim analysis will be conducted after enrollment of 50% of the planned sample size. The Haybittle-Peto stopping rule will be applied, with statistical significance for early termination defined as a *P* <.002 at the interim analysis stage. However, the final analysis will retain a 2-sided *α* level of .05.

### Monitoring Sites During the Study

The coordinating center will monitor the study and ensure compliance with the protocol, Good Clinical Practice guidelines, and relevant regulatory requirements. A checklist has been created to ensure the readiness of each site to start patient recruitment. A site initiation meeting will be held via an online platform, and study investigators from the primary center will conduct a site visit to ensure that sites are equipped and able to run the trial. In addition, the lead center will conduct regular online meetings to ensure compliance with the study protocol and the integrity of the study data.

### Disseminating the Study Results

The results of the trial will be disseminated through publication in a peer-reviewed journal so that the scientific community can use the conclusions of this study to manage patients better. Authorship will be based on contributions to the study. Following completion of the study, the principal investigator will have access to the final dataset.

### Data Analysis Plan

#### Summary Statistics

Continuous data will be expressed as the mean and SD, or the median with an IQR. Categorical data will be expressed as percentages. The independent variables in the study include both continuous and categorical factors. Continuous independent variables are age, mean arterial pressure, GCS at admission, time interval between the incident and presentation, admission blood glucose level, and GOS at discharge and on day 28. The categorical independent variables include sex, type of hanging (complete or partial), material used for hanging, presence of seizures, hanging-induced cardiac arrest, requirement for mechanical ventilation, vasopressor support, and in-hospital complications. The dependent variables consist of both continuous and categorical outcomes. The continuous dependent variable is the duration of hospital stay, while the categorical dependent variables include the proportion of patients with a good neurological outcome at discharge and on day 28, as well as the occurrence of in-hospital complications.

#### Analytical Statistics

The primary and secondary outcomes will be compared between the intervention and placebo groups using appropriate statistical tests. The proportion of patients achieving good neurological outcomes on day 28, as well as the proportion achieving good neurological outcomes at discharge, will be analyzed using the chi-square or Fisher exact test, with results expressed as relative risk estimates. Similarly, the comparison of in-hospital complications between the 2 groups will also use the chi-square or Fisher exact test, with relative risk used to quantify the effect size. For continuous outcomes, such as the duration of hospital stay, comparisons will be made using either the independent *t* test or the Mann-Whitney *U* test depending on data distribution, and the effect will be reported as an absolute difference between groups.

The primary efficacy analysis will be carried out by following the intention-to-treat principle. A per-protocol analysis will be performed as a secondary analysis. If there are missing data for the outcome at day 28, multiple imputation using chained equations will be used, based on the assumption that data are missing at random [26]. The primary analysis will also adjust for GCS stratification at baseline using a multivariable regression model. No adjustment for study site will be carried out, as all the sites have a similar profile with respect to available treatment and manpower. All statistical analyses will be carried out at a 5% level of significance, and a *P* value <.05 will be considered significant. For interim analyses alone, *P*<.002 will be applied, following the Haybittle-Peto stopping rule.

#### Subgroup Analysis

Subgroup analysis will be conducted using the same analytical methods described for the primary and secondary outcome measures, including the subgroup variable and an appropriate effect size measure with a 95% CI.

Subgroup analyses were planned based on key clinical and demographic variables. These included gender (male vs female), severity of neurological impairment as assessed by GCS (≤8 vs>8), and the type of hanging (partial vs complete). Additional subgroup variables comprised the presence or absence of seizures, the presence or absence of cardiac arrest at presentation, and admission blood glucose levels categorized as <140 mg/dL vs ≥140 mg/dL.

### Sample Size

The sample size was calculated to detect a clinically meaningful difference in the proportion of patients achieving a good neurological outcome (GOS 4‐5) at day 28 between the dexamethasone and placebo groups. In our previous retrospective study, approximately 75% of near-hanging patients with altered sensorium who received steroids achieved a good neurological outcome [17]. Using these data, the expected event rate in the intervention group was assumed to be 75% while a favorable neurological outcome rate of 55% was assumed in the placebo group, corresponding to an absolute treatment effect of 20%.

The sample size for this superiority trial with a binary outcome was calculated using a 2-sided α of 0.05% and 80% statistical power. Sample size estimation was performed using the Sealed Envelope online sample size calculator for binary superiority trials [27]. The calculated sample size was 86 participants per treatment arm. To account for an anticipated 10% attrition rate due to loss to follow-up or protocol deviations, the sample size was inflated to 96 participants per arm, resulting in a total sample size of 192 participants.

The effect size was chosen as a pragmatic compromise between clinical relevance and study feasibility. Near-hanging with altered sensorium is an uncommon emergency presentation, and, based on patient admissions at the 3 study sites, 190 to 200 eligible patients were expected to be recruited during the planned study period. For detecting a smaller treatment effect, we will require a substantially larger sample size that will not be feasible within the available study period and funding. Despite this, an absolute improvement of 20% in favorable neurological outcomes was considered clinically important because such a benefit would be expected to substantially influence clinical practice, whereas failure to demonstrate a benefit of this magnitude would provide evidence against the routine empirical use of corticosteroids in this population.

### Protocol Amendments

Several amendments were made to the study protocol to improve feasibility, clarity, and participant safety. The current version of the protocol is version 1.5, dated July 1, 2025. The inclusion window was extended from 24 hours to 48 hours following a hanging attempt, as several otherwise eligible patients were referred from nearby districts and presented beyond the original 24-hour period, leading to the loss of potential participants. This change was implemented to enhance recruitment, given the relatively low number of hanging cases.

The exclusion criterion related to corticosteroid use was corrected to address a typographical error: the intended exclusion dose was clarified as 20 mg of dexamethasone or an equivalent corticosteroid dose, rather than 24 mg. Additional exclusion criteria were introduced to explicitly exclude patients with a definite indication for, or contraindication to, steroid therapy, and to clearly state the exclusion of patients who did not provide informed consent.

Amendments were also made to the criteria for discontinuation of the study drug. While the originally planned duration of study drug administration was 3 to 5 days, a predefined tapering or early discontinuation strategy was introduced for patients who achieved rapid neurological recovery (GCS score of 15/15), particularly if this occurred by day 1, to minimize unnecessary drug exposure and ensure patient safety. Furthermore, mandatory permanent discontinuation of the study drug was specified for participants who developed a definite indication for or contraindication to steroid therapy during the trial period. All amendments were made to enhance patient safety, improve protocol clarity, and address issues encountered during recruitment.

## Results

The study commenced in October 2025 and is planned to be completed by May 2028. A total of 192 participants will be recruited. At the time of manuscript submission, patient recruitment had been initiated at the primary center (JIPMER), where 16 patients had been screened and 3 patients had been enrolled. The remaining 2 participating centers are in the process of completing the necessary regulatory and logistical requirements to initiate recruitment. Data analysis will be conducted, and final results will be reported after the conclusion of the study. The protocol has been developed in accordance with the SPIRIT 2013 recommendations, and the final trial results will be reported in accordance with the CONSORT (Consolidated Standards of Reporting Trials) 2010 statement [28].

## Discussion

### Expected Findings

The hypoxic-ischemic brain injury seen in near-hanging is associated with substantial mortality and long-term neurological disability despite advances in critical care [5,7]. The secondary brain injury determines the neurological outcome despite prompt resuscitation. Jugular venous obstruction results in flow stagnation, leading to cerebral hypoxia, which may be the initial event in hanging. Excessive cerebral perfusion of an already hypoxic brain following relief of obstruction may also contribute to cerebral edema [5,21]. Currently, there is no guideline-directed therapy for improving neurological recovery in these patients, and the management is only supportive [21].

The pathophysiology of cerebral edema in near-hanging is poorly understood. The following mechanism is proposed for hypoxia-induced cerebral edema at high altitudes and in traumatic brain injury. Hypoxia at altitude elicits neuro-hormonal (VEGF, nitric oxide, reactive cytokines, and free radicals) and hemodynamic responses, resulting in hypoxia-induced cerebral vasodilation leading to over perfusion of microvascular cerebral beds. This leads to intracranial hypertension with elevated capillary pressure and capillary leakage. The disruption of the blood-brain barrier from these stressors leads to subsequent vasogenic cerebral edema. Another theory is that hypoxia induces free radical formation, causing damage or failure of the Na+/K+ATPase pump with resultant astrocyte swelling from osmotic-oxidative stress and subsequent cytotoxic edema [29]. Similarly, hypoxia can cause cerebral edema in hanging patients. Steroids are indicated to treat vasogenic cerebral edema, whereas osmotic agents such as mannitol and hypertonic saline are preferred for the treatment of cytotoxic edema. The rationale for using dexamethasone in hanging-related cerebral edema is that it has demonstrated clinical benefit in conditions characterized predominantly by vasogenic cerebral edema, including bacterial meningitis and brain tumors [30].

There is very limited evidence for the use of corticosteroid therapy in near-hanging. The existing literature is predominantly retrospective and comprises narrative reviews with several methodological limitations [8,18,31]. Our previous retrospective study demonstrated frequent empirical use of corticosteroid therapy in routine clinical practice but could not establish treatment efficacy because of its observational design [17]. No randomized controlled trials are currently available on steroid therapy specifically for near-hanging patients. However, there are studies available on steroid therapy for traumatic brain injury that closely resemble the type of brain injury seen in near-hanging cases. The MRC CRASH trial studied corticosteroids in patients with head injury, finding a higher death rate in those given corticosteroids (25.7%) compared with placebo (22.3%). This suggests that corticosteroids should not be used routinely to treat head injuries [19].

A narrative review by Kannamani et al [21] on near-hanging described head-end elevation, sedation, maintaining normocapnia (PaCO_2_ of 30‐35 mm Hg), and osmotic diuretics (hypertonic saline, mannitol) as management for cerebral edema and did not include steroid therapy in the management. A short review on steroid therapy in near-hanging patients reported that there is no evidence for or against the use of steroids in this situation [32].

Our CHANGING trial has been designed to address this evidence gap through a multicenter, randomized, double-blind, and placebo-controlled design. Random allocation and blinding of participants, treating clinicians, and outcome assessors are expected to minimize selection and assessment bias [22,28]. The study is conducted in 3 different tertiary care hospitals, which will improve external validity by including patients across diverse intensive care settings. To enhance the reliability of the study findings, the study follows a standardized treatment protocol, predefined neurological outcome, and systematic AE reporting [22].

Our trial uses patient-centered functional outcome as the primary endpoint. The assessment of neurological recovery using the Glasgow Outcome Scale at day 28 reflects clinically meaningful recovery rather than just a physiological improvement and has been widely used in studies of traumatic brain injury [24,33]. Simultaneous assessment of AEs will aid in balancing potential neurological benefits against complications related to corticosteroids, including hyperglycemia, secondary infections, hypokalemia, and gastrointestinal bleeding [34].

### Limitations

There are a few anticipated limitations to consider; this trial is being conducted at 3 tertiary care centers in India, which may limit the generalizability of the findings to other public and private hospitals or to international populations. The sample size calculation assumed a 20% absolute difference between the treatment groups, which may be considered relatively high. This assumption was made to ensure feasibility within the constraints of the 3-year study period, available funding, and the limited number of near-hanging cases, and it represents a pragmatic compromise between feasibility and statistical power. Short-term follow-up (28 d) is also a limitation.

### Conclusions

The CHANGING trial seeks to address a major evidence gap in the management of near-hanging patients with altered consciousness, a population in whom corticosteroids are widely used despite limited supporting data. Through a rigorous multicenter, double-blind, placebo-controlled design, this study aims to determine whether dexamethasone improves neurological outcomes and safety. The results are expected to provide definitive evidence to guide clinical practice and inform future guidelines for managing near-hanging-associated cerebral injury.

## Supplementary material

10.2196/100623 Checklist 1 SPIRIT checklist

10.2196/100623 Peer Review Report 1

## Abbreviations

AE: adverse event
CONSORT: Consolidated Standards of Reporting Trials
CRF: case record form
DSMB: Data and Safety Monitoring Board
GCS: Glasgow Coma Scale
GMP: Good Manufacturing Practice
GOS: Glasgow Outcome Scale
HACE: high-altitude cerebral edema
ICMR: Indian Council of Medical Research
IGMCRI: Indira Gandhi Medical College and Research Institute
IGMCRI: Indira Gandhi Medical College and Research Institute
JIPMER: Jawaharlal Institute of Postgraduate Medical Education and Research
LAR: legally acceptable representative
PF: PaO_2_/FiO_2_
PGIMER: Postgraduate Institute of Medical Education and Research
SAE: serious adverse event
SOFA: Sequential Organ Failure Assessment
SPIRIT: Standard Protocol Items: Recommendations for Interventional Trials

## References

[1] (2021). Suicide worldwide in 2019: global health estimates. https://iris.who.int/server/api/core/bitstreams/3bd4ac79-4347-420e-b675-948d36ab3d90/content.

[2] (2024). Accidental deaths and suicides in India 2022. https://ncrb.gov.in/uploads/nationalcrimerecordsbureau/custom/adsiyearwise2022/1701611156012ADSI2022Publication2022.pdf.

[3] Adams N (1999). Near hanging. Emerg Med.

[4] Ganesan P, Jegaraj MKA, Kumar S, Yadav B, Selva B, Tharmaraj RGA (2018). Profile and outcome of near-hanging patients presenting to emergency department in a tertiary care hospital in South India - a retrospective descriptive study. Indian J Psychol Med.

[5] Salim A, Martin M, Sangthong B, Brown C, Rhee P, Demetriades D (2006). Near-hanging injuries: a 10-year experience. Injury.

[6] Dorfman JD (2023). Near hanging: evaluation and management. Chest.

[7] Matsuyama T, Okuchi K, Seki T, Murao Y (2004). Prognostic factors in hanging injuries. Am J Emerg Med.

[8] Vander Krol L, Wolfe R (1994). The emergency department management of near-hanging victims. J Emerg Med.

[9] Sekhon MS, Ainslie PN, Griesdale DE (2017). Clinical pathophysiology of hypoxic ischemic brain injury after cardiac arrest: a “two-hit” model. Crit Care.

[10] Stokum JA, Gerzanich V, Simard JM (2016). Molecular pathophysiology of cerebral edema. J Cereb Blood Flow Metab.

[11] Klatzo I (1987). Pathophysiological aspects of brain edema. Acta Neuropathol.

[12] Jha RM, Kochanek PM, Simard JM (2019). Pathophysiology and treatment of cerebral edema in traumatic brain injury. Neuropharmacology.

[13] Unterberg AW, Stover J, Kress B, Kiening KL (2004). Edema and brain trauma. Neuroscience.

[14] Cook AM, Morgan Jones G, Hawryluk GWJ (2020). Guidelines for the acute treatment of cerebral edema in neurocritical care patients. Neurocrit Care.

[15] Luks AM, Auerbach PS, Freer L (2019). Wilderness Medical Society clinical practice guidelines for the prevention and treatment of acute altitude illness: 2019 update. Wilderness Environ Med.

[16] Hackett PH, Yarnell PR, Weiland DA, Reynard KB (2019). Acute and evolving MRI of high-altitude cerebral edema: microbleeds, edema, and pathophysiology. AJNR Am J Neuroradiol.

[17] Ramadoss R, Sekar D, Rameesh M, Saibaba J, Raman D (2023). Clinical profile, corticosteroid usage and predictors of mortality in near-hanging patients: a five-year, single-center retrospective study. Indian J Crit Care Med.

[18] Carney N, Totten AM, O’Reilly C (2017). Guidelines for the management of severe traumatic brain injury, fourth edition. Neurosurgery.

[19] Edwards P, Arango M, Balica L (2005). Final results of MRC CRASH, a randomised placebo-controlled trial of intravenous corticosteroid in adults with head injury-Outcomes at 6 months. Lancet.

[20] Gandhi R, Taneja N, Mazumder P (2011). Near hanging: early intervention can save lives. Indian J Anaesth.

[21] Kannamani B, Sahni N, Bandyopadhyay A, Saini V, Yaddanapudi LN (2024). Insights into pathophysiology, management, and outcomes of near-hanging patients: a narrative review. J Anaesthesiol Clin Pharmacol.

[22] Chan AW, Tetzlaff JM, Altman DG (2013). SPIRIT 2013 statement: defining standard protocol items for clinical trials. Ann Intern Med.

[23] Chan AW, Tetzlaff JM, Gøtzsche PC (2013). SPIRIT 2013 explanation and elaboration: guidance for protocols of clinical trials. BMJ.

[24] Jennett B, Bond M (1975). Assessment of outcome after severe brain damage. Lancet.

[25] National Cancer Institute (NCI) (2017). Common terminology criteria for adverse events (CTCAE) version 5.0. https://dctd.cancer.gov/research/ctep-trials/for-sites/adverse-events/ctcae-v5-5x7.pdf.

[26] Sterne JAC, White IR, Carlin JB (2009). Multiple imputation for missing data in epidemiological and clinical research: potential and pitfalls. BMJ.

[27] Power calculator for binary outcome superiority trial. Sealed Envelope.

[28] Schulz KF, Altman DG, Moher D, CONSORT Group (2010). CONSORT 2010 statement: updated guidelines for reporting parallel group randomised trials. BMJ.

[29] McGowan J, Huecker MR, Vincent AL (2024). StatPearls.

[30] Vecht CJ, Hovestadt A, Verbiest HB, van Vliet JJ, van Putten WL (1994). Dose-effect relationship of dexamethasone on Karnofsky performance in metastatic brain tumors: a randomized study of doses of 4, 8, and 16 mg per day. Neurology.

[31] Coombs AE, Ashton-Cleary D (2023). Hanging and near-hanging. BJA Educ.

[32] Jenner R (2008). Towards evidence based emergency medicine: best BETs from the Manchester Royal Infirmary. BET 5. Steroids in attempted hanging. Emerg Med J.

[33] Wilson JT, Pettigrew LE, Teasdale GM (1998). Structured interviews for the Glasgow Outcome Scale and the extended Glasgow Outcome Scale: guidelines for their use. J Neurotrauma.

[34] Roberts I, Yates D, Sandercock P (2004). Effect of intravenous corticosteroids on death within 14 days in 10008 adults with clinically significant head injury (MRC CRASH trial): randomised placebo-controlled trial. Lancet.

