# Supplementary material for: Efficacy of Corticosteroid Therapy on Neurological Outcomes in Near-Hanging Patients With Altered Sensorium: Protocol for a Multicenter, Double-Blinded, Placebo-Controlled, Randomized Trial (CHANGING Trial)
Source: JMIR Res Protoc. 2026 Aug 25;15:e100623. doi: 10.2196/100623 (PMC13504622; doi:10.2196/100623)
Supplement: Supplementary file 1 [file resprot-v15-e100623-s002.pdf]

Gmail **Ramadoss R <ramadoss2912@gmail.com>**

ICMR: Proposal Id IIRPSG-2024-01-00787 result (no-reply to this email)

4 messages

---

no-reply@hq.icmr.org.in <no-reply@hq.icmr.org.in> Fri, Nov 1, 2024 at 12:28 PM
To: Dr Ramadoss R <ramadoss2912@gmail.com>
Cc: ICMR-ePMS <po.epms@icmr.gov.in>, ICMR-ePMS <sudipto.roy@icmr.gov.in>, ICMR-ePMS <no-reply@hq.icmr.org.in>

Dear Dr Ramadoss R,

This is with reference to your proposal Id “**IIRPSG-2024-01-00787**”, titled “**Corticosteroid therapy for near-hanging patients: A double-blinded, multi-centre, placebo-controlled, randomized controlled trial**”.

We are pleased to inform you that your project has been approved, subject to satisfactory response to the following technical and budgetary comments (as applicable).

Technical Comments: Ethics: Since it is already a practice and investigators suspect steroids improve the neurological outcomes in observational research plesase justify why it is ethical to not provide steroid to control group. The justification for this study is that an RCT will generate evidence that can decide if guidelines can be developed: Please specify what good neurological outcome is and how it is assessed. Also specify time point at which the primary outcome is assessed. If the study is recommended for funding, there must be co-investigators from Emergency Medicine departments at all three study sites, within two weeks of receipt of this email, please upload a revised proposal addressing the technical comments. For ease of review do highlight the revisions.

Budgetary Comments: Detailed budget justification needed, PI to prepare a justification of budget and response to budgetary comments for a VC meeting with Budget Review Committee to be held within the next two weeks of receipt of the email.

We would also ask you to urgently arrange for all the CODAL formalities to be completed as per the current Guidelines for Extramural Research Programme of ICMR. ***Please submit for IEC/ regulatory approvals (as applicable) immediately.***

If the documents pertaining to CODAL formalities are not uploaded in ICMR-ePMS portal within the stipulated period (not later than 3 months of this email),the approval may be cancelled.

For any clarification/s on the technical or budgetary comments, please contact "Dr. Sudipto Roy (sudipto.roy@icmr.gov.in)"

Important Note: This is an automated email. Kindly refrain from replying to this email.

Warm Regards
Program Officer

ICMR
INDIAN COUNCIL OF MEDICAL RESEARCH
Serving the nation since 1911

e-Project Management System
Indian Council of Medical Research
Department of Health Research
Ministry of Health & Family Welfare
Government of India
Ansari Nagar, New Delhi - 110029, India
www.icmr.gov.in

</html

Ramadoss R <ramadoss2912@gmail.com> Wed, Nov 6, 2024 at 1:48 PM
To: manuayyan@hotmail.com
